# Genome Sequence of the Lager-Brewing Yeast *Saccharomyces* sp. Strain M14, Used in the High-Gravity Brewing Industry in China

**DOI:** 10.1128/genomeA.01194-17

**Published:** 2017-10-26

**Authors:** Chunfeng Liu, Qi Li, Chengtuo Niu, Feiyun Zheng, Yongxian Li, Yun Zhao, Xiangsheng Yin

**Affiliations:** a Key Laboratory of Industrial Biotechnology, Ministry of Education, Jiangnan University, Wuxi, Jiangsu Province, People’s Republic of China; b Lab of Brewing Science and Engineering, Jiangnan University, Wuxi, Jiangsu Province, People’s Republic of China; c Cargill Malt, Wayzata, Minnesota, USA

## Abstract

Lager-brewing yeasts are mainly used for the production of lager beers. Illumina and PacBio-based sequence analyses revealed an approximate genome size of 22.8 Mb, with a GC content of 38.98%, for the Chinese lager-brewing yeast *Saccharomyces* sp. strain M14. Based on *ab initio* prediction, 9,970 coding genes were annotated.

## GENOME ANNOUNCEMENT

Beer is one of the most consumed alcoholic beverages, and China’s beer production has been the largest in the world for more than 10 years (1). In China, the majority of the beer products are light beer using high-gravity brewing (HGB) with dilution technology (2). Brewing yeasts play important roles during HGB beer fermentation. To satisfy the need of the HGB process, the brewing yeast M14, with suitable characteristics for fermentation in high-gravity wort, was obtained, and it is now widely used in the Chinese brewing industry (3). In this study, we aimed to sequence the genome of the brewing yeast *Saccharomyces* sp. strain M14 and to gain detailed insights into its genomic features. To our knowledge, this is the first report of the genome sequence of an industrial lager-brewing yeast from China.

The genome DNA of M14 was extracted using the Yeast genomic DNA kit (CW0569S; CWBio, Beijing, China). Genomic DNA library construction and draft genome sequencing were performed in Shanghai Personal Biotechnology Co., Ltd. (Shanghai, China) using the Illumina MiSeq system. Quality control procedures removed DNA spike-in, artifacts, and ambiguous or low-quality reads (4, 5). Paired ends having at least 90% of bases with a quality score greater than or equal to Q_20_ were filtered before assembly. These sequences were assembled *de novo* using the SPAdes (version 3.7.1) software package (6), and the results were calibrated using the Pilon software. To predict genes in the M14 genome, we used a Web server for gene prediction in eukaryotes, AUGUSTUS (7), which is specifically trained for this genome, and one homology-based gene predictor, Exonerate (8). Using a heuristic approach implemented in a homemade pipeline, we combined all predicted gene models to produce a nonredundant set of genes, in which a single best-gene model per locus was selected on the basis of sequence similarity to known proteins. We annotated and classified genes according to four databases: Gene Ontology (GO), Kyoto Encyclopedia of Genes and Genomes (KEGG), Evolutionary Genealogy of Genes: Nonsupervised Orthologous Groups (eggNOG), and Swiss-Prot.

The resulting genome sequence of M14 has an estimated size of 22.84 Mb and a GC content of 38.98%. Three sequencing libraries (PE 400, PE 5K, and S-10K) were sequenced, resulting in 28,064,706 paired reads (8,167,798,336 bp), 18,906,632 paired reads (4,472,881,298 bp), and 2,922,174 paired reads (6,712,756,353 bp), respectively.

These reads were assembled in 133 scaffolds, with a scaffold *N*_50_ value of 575,172 bp. This representative set included 9,970 protein-coding genes. The majority (66%) of the predicted genes contained multiple exons, with an average of 1.00 exons per gene. The average gene density, similar to that of larger scaffolds, was 1.0 kb per gene. Proteins including molecular fuction proteins, cellular component proteins, and biological process proteins were annotated. We assigned the Swiss-Prot database to 9,936 (99.66%) of the predicted M14 proteins. We also assigned 6,560 (65.80%) proteins to the nonredundant (NR) database, 5,124 (51.39%) proteins to the eggNOG database, 3,724 (37.35%) proteins to the KEGG database, and 2,159 (21.65%) proteins to the GO database.

### Accession number(s).

This whole-genome shotgun project has been deposited at GenBank under the accession number MVPU00000000. The version described in this paper is the first version, MVPU01000000.
